# Ablating Aspiration Needle Tract Prior to Microwave Ablation Can Improve Therapeutic Outcomes for Predominantly Cystic Thyroid Nodules

**DOI:** 10.3389/fendo.2021.752822

**Published:** 2021-09-24

**Authors:** Haizhen Yang, Yanwei Chen, Baoding Chen, Shuangshuang Zhao, Zheng Zhang, Keke Wang, Zheming Chen, Huahui Feng, Maohui An

**Affiliations:** Department of Medical Ultrasound, Affiliated Hospital of Jiangsu University, Zhenjiang, China

**Keywords:** intracystic hemorrhage, ablating aspiration needle tract, microwave ablation, predominantly cystic nodules, volume reduction rate

## Abstract

**Purpose:**

To investigate whether ablating the aspiration needle tract could improve the safety and efficacy of ultrasound-guided microwave ablation (MWA) for predominantly cystic thyroid nodules.

**Materials and Methods:**

This retrospective study evaluated 41 predominantly cystic thyroid nodules that underwent MWA between June 2017 and August 2019. The nodules were stratified by different procedures into two groups: the aspiration needle tract was ablated before cyst fluid aspiration and MWA when treating 26 nodules in Group A, while the other 15 nodules in Group B underwent MWA directly after cyst fluid aspiration. Baseline characteristics, intervention time, hospital stays, nodules with intraoperative intracystic hemorrhage, and postoperative complications were compared between the two groups. Volume, volume reduction rate (VRR), compressive score (CS), and aesthetic score (AS) were evaluated during follow-up.

**Results:**

Both groups achieved decreases in volume, CS, and AS, as well as an increase in VRR. The volumes and VRRs in Group A at 1, 3, 6, and 12 months were significantly smaller and greater than those in Group B (p < 0.001). The incidence of intraoperative intracystic hemorrhage in Group A was significantly lower than that in Group B (p=0.035). Compared to Group B, hospital stays were much shorter in Group A (p=0.040). There were no significant differences in intervention time, cystic fluid volume or postoperative complications.

**Conclusion:**

Aspiration needle tract ablation dramatically reduces the incidence of intraoperative intracystic hemorrhage and markedly improves the efficacy of MWA for predominantly cystic thyroid nodules.

## Introduction

The development of imaging technology, especially the application of high-resolution ultrasound (US), has improved the diagnosis of thyroid nodules (TNs) in recent decades. The detection rate of cystic or predominantly cystic nodules (cystic component ≥50%) accounts for approximately 15–25% of TNs (1, 2). The American Thyroid Association (ATA) proposed that regular follow-up and specific treatments are required for predominantly cystic thyroid nodules due to compression symptoms, signs of malignant tendency, or patient concerns (3, 4). At present, percutaneous ethanol ablation (EA) is recommended as the first-line treatment option for predominantly cystic thyroid nodules; however, the efficacy may be insufficient for nodules with solid components > 20% (5–7). MWA is a method of treating TNs worldwide (8–12), and most patients achieve good prognosis. Nevertheless, intracystic hemorrhage occurred frequently in the US-guided MWA process for TNs, which may impair the therapeutic efficacy, leading to the failure of treatment or excision. Blood vessels on the capsule wall bleed due to the penetration of the puncture needle. The tension of the nodule is reduced after aspiration, resulting in relief of the pressure at the bleeding point from the puncture needle. Bleeding during the MWA process is accelerated (13). In our clinical practice, a newly proposed procedure of MWA that has been applied is as follows: First, the optimal needle track for aspiration should be selected. Then, the ablation needle is inserted, and the tract ablated for a few seconds. Immediately afterward, the ablation needle is withdrawn and the puncture needle is inserted along this path to aspirate the cystic fluid. Finally, the ablation needle is reinserted to perform MWA for the entire nodule. Prior to ablation of the needle tract, the punctured blood vessel on the capsule wall is occluded. Therefore, when the puncture needle enters the nodule to aspirate the cystic fluid along this path, the risk of intraoperative hemorrhage is greatly reduced. As expected, according to our clinical observations, this ablation resulted in a reduction in intraoperative intracapsular hemorrhage nodules. Regrettably, to date, there have been few studies focusing on and reporting such issues.

The aim of this study was to investigate whether ablating the aspiration needle tract could reduce the incidence of intraoperative intracystic hemorrhage and postoperative complications and improve the efficacy of US-guided MWA for predominantly cystic thyroid nodules.

## Materials and Methods

This study was designed as a retrospective observational study and approved by the Ethics Committee of the Affiliated Hospital of Jiangsu University (SWYXLL20190225–2). Written informed consent was obtained from each of the patients before MWA, as long as the patients’ identities were protected.

### Study Participants

**Figure 1:**
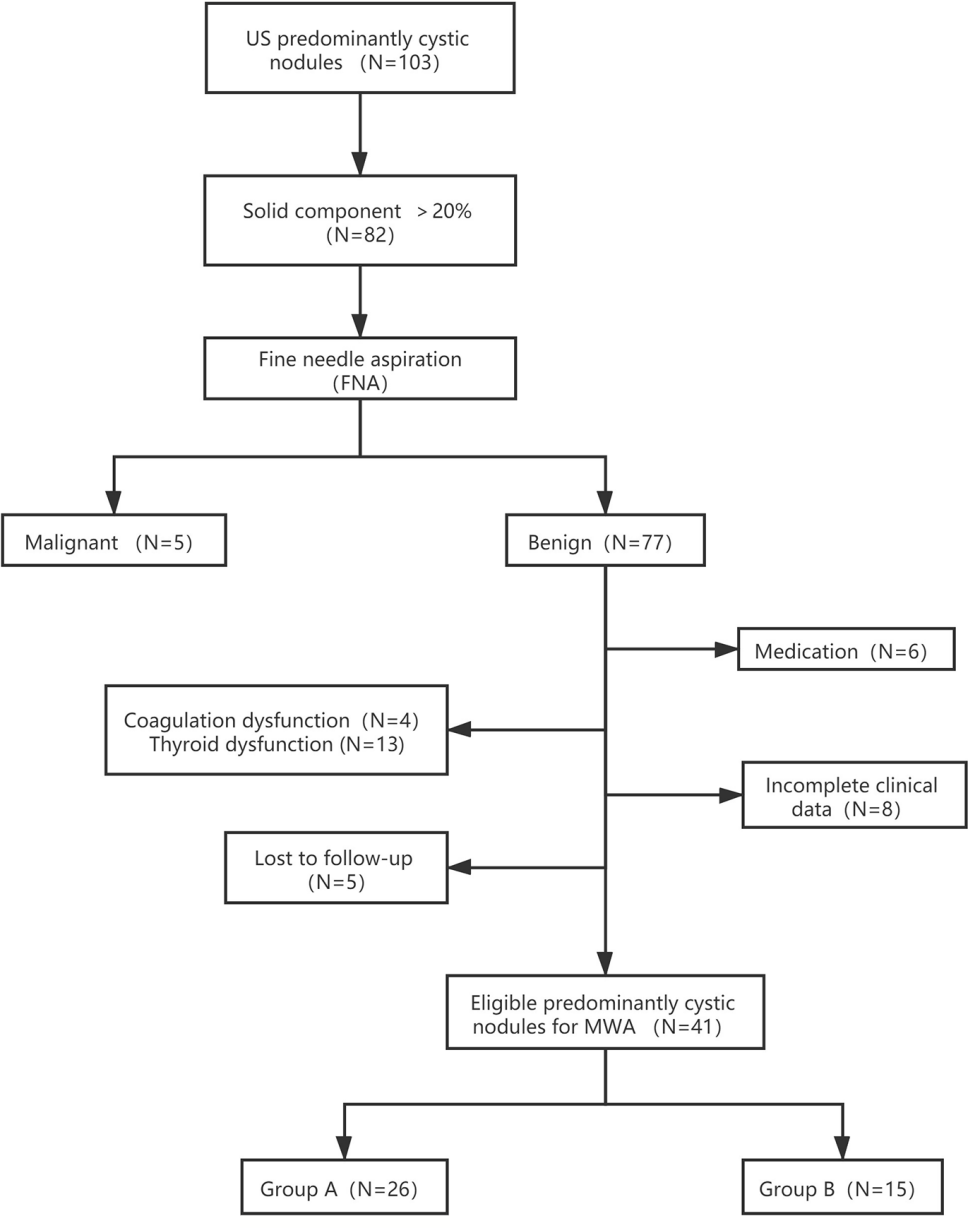
Flowchart of study participants included and excluded in the study. Group A: Aspiration neddle tract was ablated prior to cyst fluid aspiration. Subsequently, the MWA for the entire nodule was proformed. Group B: MWA was performed directly after aspiration of internal cyst fluid.

A total of 41 predominantly cystic thyroid nodules in 38 patients (8 males and 30 females; mean age: 51.37 ± 13.02 years old; range: 24–72 years old) treated with MWA at the Affiliated Hospital of Jiangsu University from June 2017 to August 2019 were included (**Figure 1**).

### Equipment

MyLabTwice (Esaote, Italy) with probes LA523 and LA332 was utilized to perform ultrasonic examination. SonoVue (Bracco, Italy), a contrast agent mainly composed of sulfur hexafluoride microbubbles, was employed for contrast-enhanced ultrasound (CEUS) examinations. An ECO-100E microwave generator system (ECO, China) equipped with a 16-gauge disposable microwave ablation needle coupled with an antenna was used for the ablation process. The output power was set to 35 W while the frequency was 2450 MHz, and an internally cooled needle antenna with normal saline for cold circulation fluid was used.

### Inclusion and Exclusion Criteria

All nodules were pathologically confirmed as benign *via* US-guided fine needle aspiration (FNA) cytology. The 41 nodules were selected based on the following inclusion criteria (1): aged ≥18 years; (2) maximum nodule diameter ≥3 cm; (3) solid component of the nodule >20%; (4) presence of subjective symptoms such as neck swelling, pain, and foreign sensation; (5) patients with nodule anxiety who have a therapeutic appeal; and (6) overall poor condition that cannot tolerate surgery or unwillingness to undergo surgery due to aesthetic needs. The exclusion criteria were as follows: (1) coagulation dysfunction, thyroid dysfunction, abnormal blood pressure, blood glucose, or platelets; (2) US results showing highly malignant nodules, although the FNA results showed benign nodules; (3) incomplete clinical data; (4) recent use of aspirin, warfarin, and other anticoagulants; and (5) cystic component of the nodule >80%.

### MWA Procedure

Based on the size, location, blood flow, and CEUS results, the best aspiration and ablation track were determined. Each patient was placed in the supine position with a fully exposed neck before the operator disinfected the skin, locally anesthetized, and opened venous access for CEUS and intraoperative rescue. Lidocaine (2%) (5 ml) was infused into the surrounding thyroid capsule to reduce pain stimulation prior to aspiration and ablation. A mixture of lidocaine (10 ml) and 0.9% normal saline (20 ml) was injected into the surrounding thyroid capsule to create a “liquid isolating region”, intending to protect the adjacent trachea, nerve, and arteriovenous.

**Figure 2:**
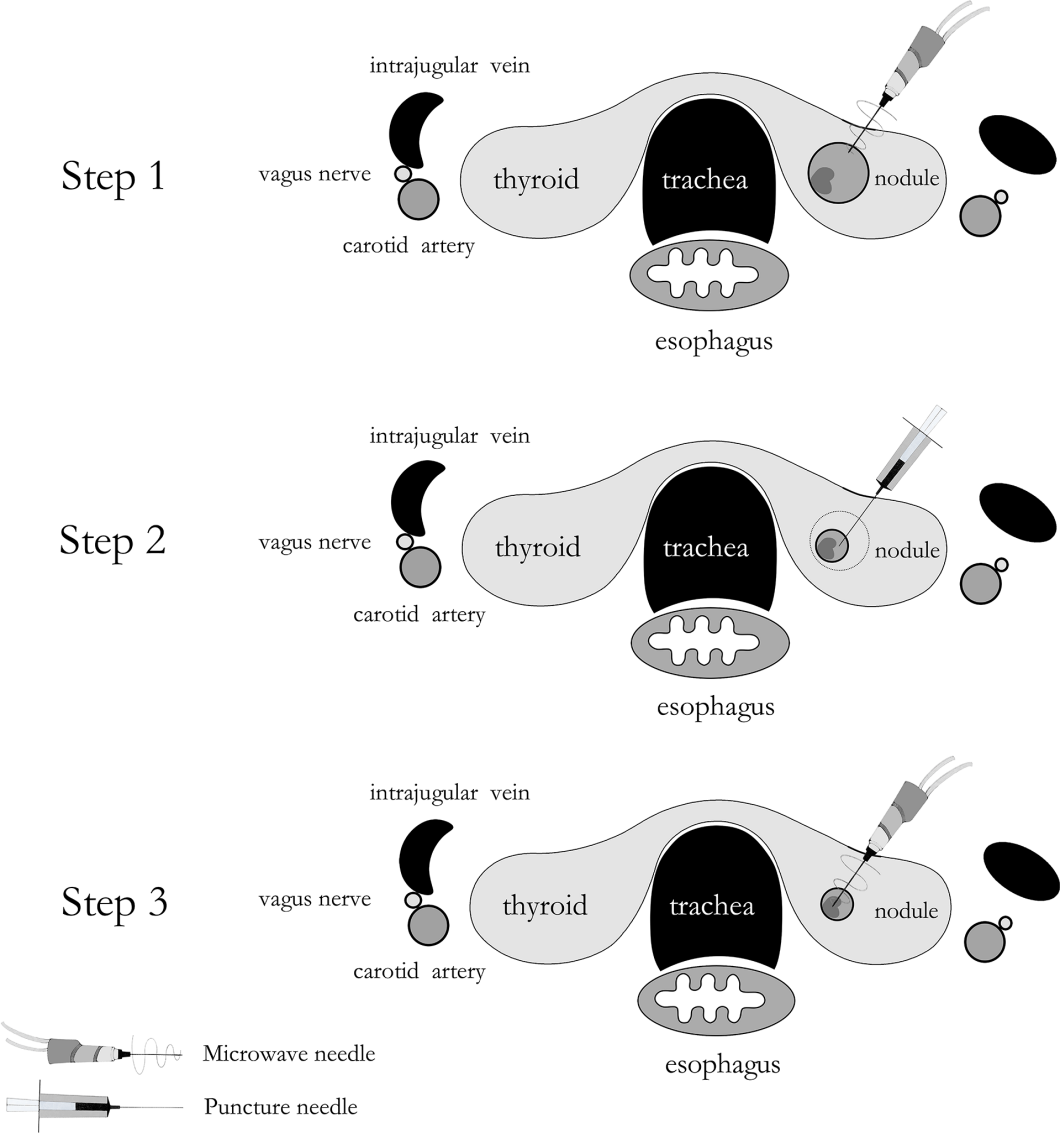
A schematic drawing of ablating aspiration needle tract before microwave ablation for predominantly cystic thyroid nodules.

**Figure 3:**
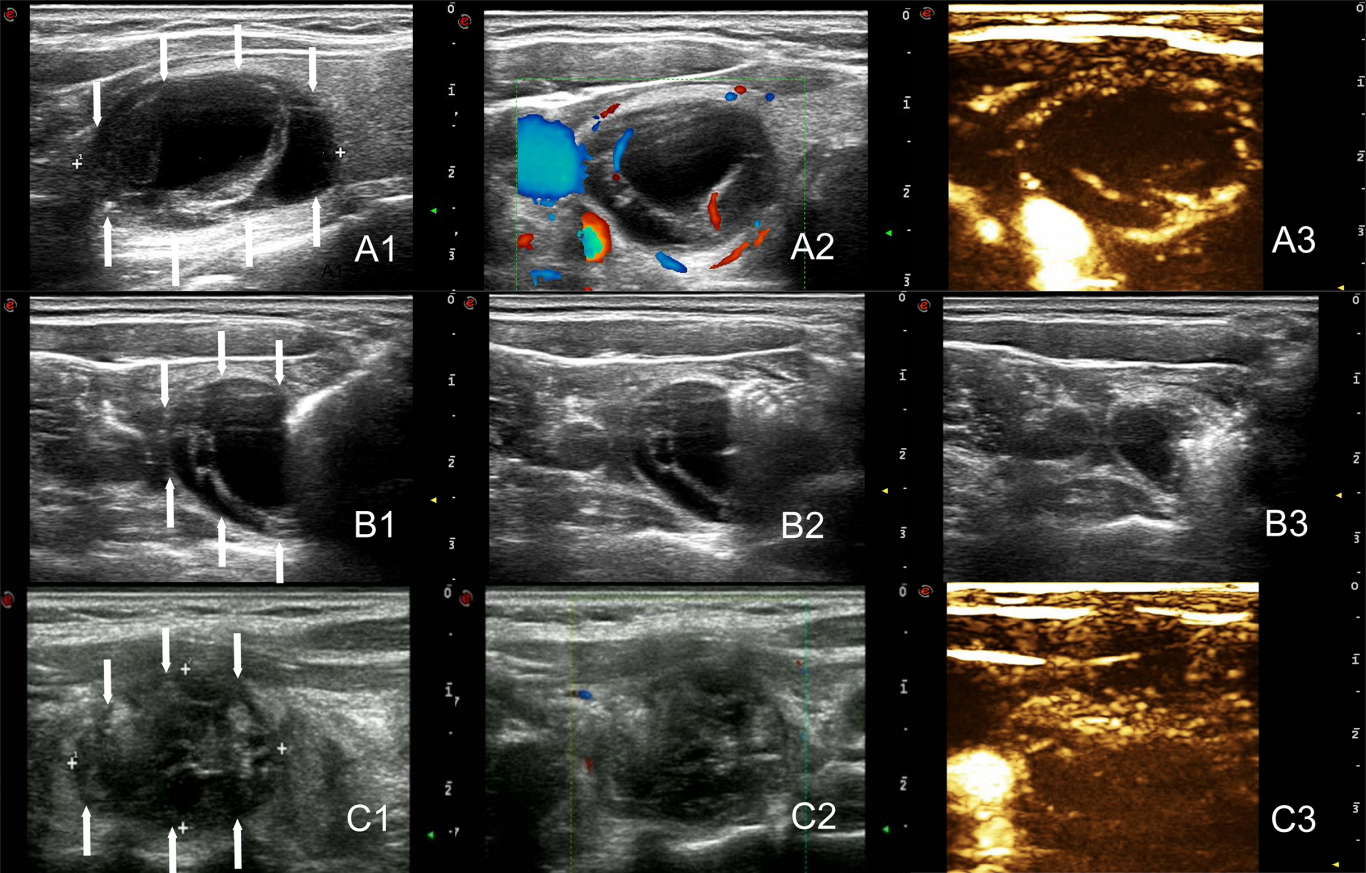
The arrows point to the nodules. Two-dimensional ultrasound, CDFI and CEUS images before treatment of the nodule (Volume = 9.1ml, **A1–A3**). Images of the tract ablation in group A. One needle tract ablated prior to cyst fluid aspiration, followed by an additional ablation of the entire nodule. **(B1–B3)**. Two-dimensional ultrasound, CDFI and CEUS images immediately after the ablation of the nodule (Volume = 4.4ml, **C1–C3**).

The MWA methods for 26 nodules in Group A were detailed as follows: The microwave needle was pinned accurately to the capsule wall along the optimal aspiration and ablation needle tract, and the route was ablated first with a power output of 35 W. Then, a 20 ml syringe was inserted into the nodule along the ablation path described above, and the internal fluid was aspirated. Finally, MWA was performed for the entire nodule (**Figure 2**). The two-dimensional ultrasound, CDFI, and CEUS images during the procedure are shown in **Figure 3**. The internal fluid of 15 nodules in Group B was first aspirated, and then MWA was performed with little fluid remaining.

Nodule volume significantly decreased after aspiration of internal fluid in both groups. The ablation process was carried out under dynamic ultrasonic monitoring. The procedure was conducted by the “moving-shot” technique (14). The area adjacent to the capsule wall and the solid part of the nodules were given more attention during MWA. With the release of microwave energy, the echo from the microwave needle was incrementally enhanced, and ablation was completed until CEUS showed no blood flow signal in the nodules.

When intracystic hemorrhage occurred, the operator stopped the procedure. Under US monitoring, the shrunken nodules gradually expanded. Sometimes enlargement was visible to the naked eye, and even in some special cases, bleeding points and blood flow could be seen. Bleeding was confined to the cysts and did not affect other tissues (15). Once this happened, the puncture point was compressed for 3–5 min to staunch bleeding. If the bleeding stopped, the previous aspiration and ablation steps were repeated. If bleeding persisted or recurred after the procedure, aspiration, observation, searching for the bleeding point, and ablation were performed.

Intracystic hemorrhage was observed and recorded in the treatment. After the treatment, all the patients compressed their necks for 5 minutes and were kept under observation for more than 2 h in case of potential complications. Finally, we recorded and analyzed the intervention time.

### Group Criteria

Twenty-six thyroid nodules with ablation of the needle tract before aspiration were classified as Group A. Fifteen thyroid nodules were directly ablated after aspiration, and they were classified as Group B.

### Preoperative Assessment

The initial maximum diameter of the nodules and two vertical diameters were measured under US, and the volume was calculated according to the following formula: V=length × width × depth × π/6 (3). The proportion of the cystic component in the nodules and the location of the nodules were evaluated. The general information of all the patients attained at the first US examination included age, sex, compressive score (CS), and aesthetic score (AS). The CS was self-measured by the patient with a 10 cm visual analog scale (0–10). Before treatment, the physicians recorded the AS: (1) no palpable mass; (2) no cosmetic problem but a palpable mass; (3) cosmetic problem only on swallowing, and (4) readily detected cosmetic problem (9).

In addition, coagulation function, thyroid function, and serum glucose (GLU) were assessed within one week before MWA. Color Doppler flow imaging (CDFI) is currently widely used to perform Rosario (16) classification at the first US for evaluating the vascularity of TNs. In our work, grade 0–1 was defined as scanty blood supply, and grade 2 was abundant blood supply. US, US-guided FNA, and MWA of predominantly cystic thyroid nodules were performed by experienced radiologists, while the cytological results were given by the cytopathology expert. The above general, clinical, and ultrasonic characteristics were compared between the two groups.

### Follow-Up After MWA of All Thyroid Nodules

After MWA, ultrasound examination of the laryngea was performed immediately. Similarly, postoperative thyroid function needed to be evaluated. Thyroid US was performed at 1, 3, 6, and 12 months as well as every six months after ablation. The volume, VRR, CS, and AS were compared during the follow-up period. The VRR was calculated using the following equation: VRR (%) = (initial volume - follow-up volume)/initial volume ×100% (17).

### Follow-Up After MWA of Multiple Comparisons Between the Two Groups

Concurrently, the differences in hospital stays, volume, VRR, number of nodules with intraoperative intracystic hemorrhage, and postoperative complications (major complications including voice changes, skin burning, infection, esophageal injury or tracheal injury, and minor complications including hematoma, vomiting, skin burns, and mild pain) were analyzed between the two groups.

### Statistical Analysis

All statistical analyses were performed using SPSS version 26.0. Quantitative data are expressed as the means ± standard deviation. The comparison was performed using an independent sample t-test for continuous variables and the χ2 test for categorical variables. The Wilcoxon test was used to compare nodule volume and VRR before and after MWA. The volume and VRR of predominantly cystic nodules after treatment were compared by the Mann–Whitney U-test. A P value less than 0.05 was considered statistically significant.

## Results

### Clinical and US Characteristics Before MWA

The clinical profiles of the included patients in the two groups are shown in **Table 1**. There was no significant difference in sex, age, coagulation function, thyroid function, CS, AS, or GLU between the two groups (all p >0.05). Notably, the initial volume, blood supply, location, and longest diameter of the nodules were not significantly different between the two groups (all p >0.05).

**Table 1:** Comparison of general clinical and ultrasound characteristics between the two groups.

	Group A	Group B	P- value
Patients	N = 23	N = 15	
Gender (female)	17/23	13/15	0.440
Age (years)	54.52 ± 13.96	46.53 ± 10.00	0.064
Coagulation function			
PT	10.77 ± 0.64	10.60 ± 0.54	0.403
APTT	25.80 ± 1.66	25.15 ± 1.96	0.278
Thyroid function			
TSH	1.53 ± 0.56	1.71 ± 0.73	0.374
TgAb	13.51 ± 5.80	18.75 ± 11.78	0.076
TPOA	11.46 ± 4.08	13.19 ± 4.17	0.212
GLU	5.40 ± 0.51	5.38 ± 0.50	0.933
CS	4.26 ± 1.42	3.40 ± 1.50	0.083
AS	3.42 ± 0.73	3.60 ± 0.63	0.477
Nodules	N = 26	N = 15	
Longest diameter (mm)	38.04 ± 7.79	38.40 ± 5.69	0.876
Pre-treatment Volume (ml)	16.06 ± 10.99	13.60 ± 5.95	0.787
Nodule blood supply type %			0.975
Scanty blood supply	14 (53.7)	8 (53.3)	
Abundant blood supply	12 (46.3)	7 (46.7)	
Location (%)			0.536
Left	13 (50)	9 (60.0)	
Right	13 (50)	6 (40.0)	

Values are presented as mean ± SD, n/N or n (%).

GLU, serum glucose; CS, compressive score; AS, aesthetic score; PT, plasma prothrombin time; APTT, activated partial thromboplastin time; TSH, thyroid stimulating hormone; TPOAb, anti-thyroid peroxidase antibody; TgAb, thyroglobulin antibodies.

### Multiple Comparisons Between the Two Groups During and After MWA

The volume of cystic fluid aspirated and the intervention time of the nodules in the two groups (all P > 0.05) were observed to be statistically insignificant. In addition, six nodules in Group B experienced intracystic hemorrhage during MWA, resulting in an incidence of 40.0% (6/15), which was higher than that in Group A, with an incidence of 8.7% (2/26) amidst the differences being statistically significant (P = 0.035). However, there was no significant difference in minor complications between the two groups after MWA (P =0.550). Additionally, no serious complications were observed in either group. Patients in Group A had fewer hospital stays than those in Group B, but the difference was not statistically significant (P =0.06) (**Table 2**).

**Table 2:** Comparison of the clinical characteristics between the two groups during and after MWA.

	Group A	Group B	*P*-value
Patients	N = 23	N = 15	
Hospital stays(days)	1.13 ± 0.34	1.40 ± 0.51	0.085
Complication (%)			
Minor complication	1 (4.3)	2 (13.3)	0.550
Major complication	0	0	/
Nodules	N = 26	N = 15	
Intervention time (seconds)	301.58 ± 227.74	314.33 ± 121.11	0.842
Cystic fluid volume (ml)	13.23 ± 12.90	12.00 ± 9.52	0.749
No. of intracystic hemorrhaging during MWA	2/26	6/15	0.035

Values are presented as mean ± SD, n/N or n (%).

### Comparison of Intraoperative Intracystic Hemorrhaging Between the Two Groups of Nodules With Abundant Blood Supply

In the MWA process, intraoperative intracystic hemorrhage occurred in 19.5% (8/41) of nodules. Among the 12 nodules with abundant blood supply in Group A, intraoperative intracystic hemorrhaging accounted for only 8.3%. However, among the seven nodules with abundant blood supply in Group B, intraoperative intracystic hemorrhaging accounted for 57.1%. Compared with the WMA procedure of Group B, the procedure of Group A reduced the incidence of intraoperative intracystic hemorrhaging, although there was no significant difference (**Table 3**, P = 0.073).

**Table 3:** Comparison of intraoperative intracystic hemorrhaging between two groups of nodules with rich blood supply.

	Group A (N = 12)	Group B (N = 7)	*P*-value
Nodules with intracystic hemorrhage	1 (8.3)	4 (57.1)	0.073
Nodules without intracystic hemorrhage	11 (91.7)	3 (42.9)	

### Comparison of the Volume and VRR Between the Two Groups

**Figure 4:**
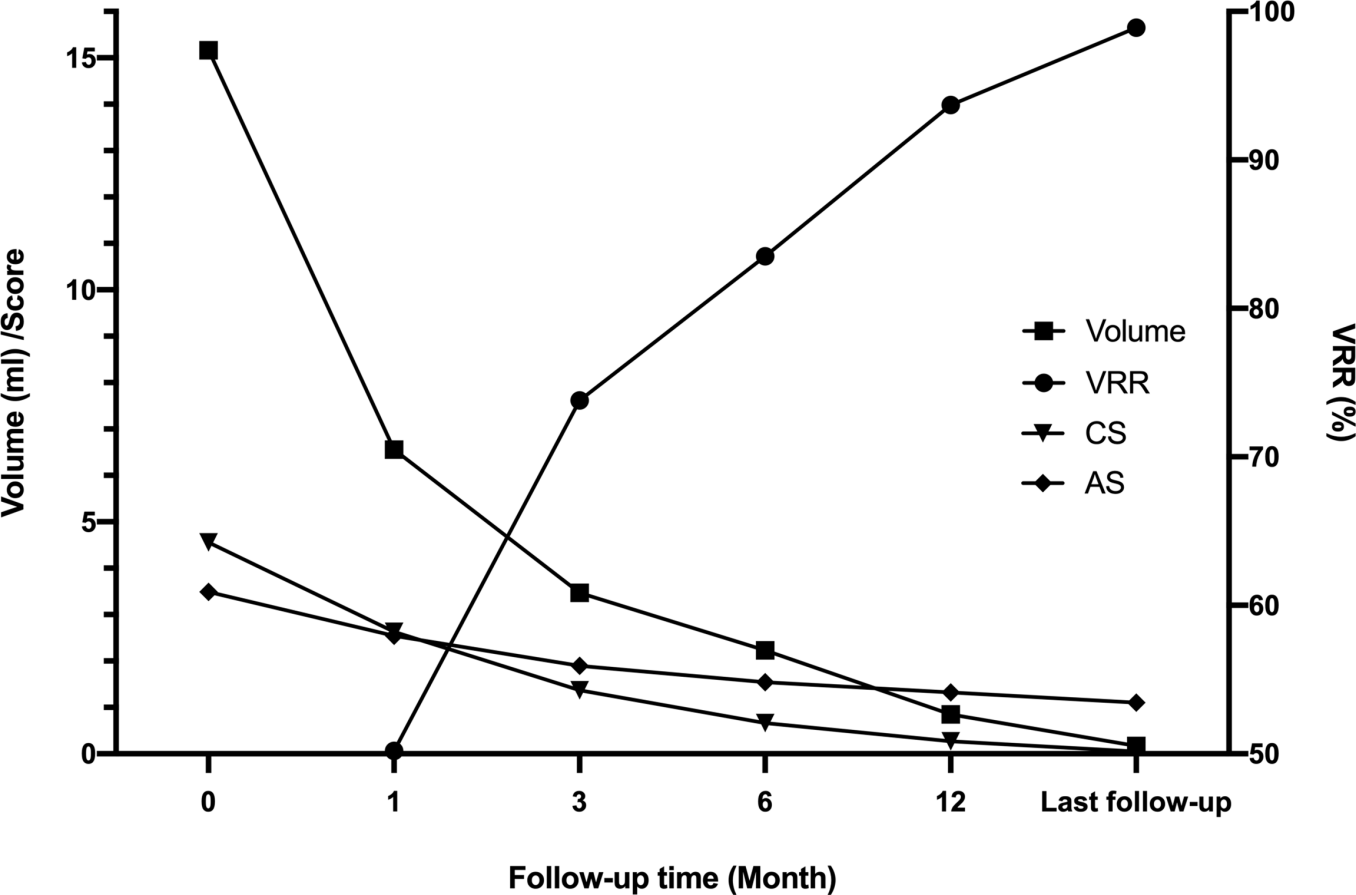
Multiple changes in the whole group before ablation and after follow-up. After MWA, the nodule volume gradually decreased, and the VRR gradually increased. The patient’s CS and AS decreased. CS, compressive score; AS, aesthetic score; VRR, volume reduction rate.

The mean volume of nodules in Group A was 16.06 ± 10.99 ml before MWA, 1.24 ± 1.45 ml at 6 months, and 0.03 ± 0.07 ml at the last follow-up. The estimated mean VRR at 1, 3, 6, and 12 months was 59.7%, 64.4%, 92.0%, and 98.2%, respectively. Comparatively, the mean volume of nodules in Group B was 13.60 ± 5.95 ml before MWA, 3.95 ± 2.67 ml at 6 months, and 0.43 ± 0.52 ml at the last follow-up. The estimated means of VRR at 1, 3, 6, and 12 months were 33.7%, 55.4%, 68.7%, and 85.9%, respectively. The decreasing volume of nodules in Group A was significantly higher than that in Group B. Concurrently, the VRR of nodules in Group A was significantly higher than that in Group B (**Table 4**, all p < 0.01). After MWA, the nodule volume and the patient’s CS and AS decreased while the VRR increased (**Figure 4**).

**Table 4:** Comparison of MWA curative effect between two groups.

	Group A (26)		Group B (15)		*P*-value
	Volume (ml)	VRR (%)	Volume (ml)	VRR (%)	
Before MWA	16.06 ± 10.99	/	13.60 ± 5.95	/	0.787
1month after MWA	5.54 ± 4.75	59.7	8.34 ± 3.81	33.7	<0.001
3month after MWA	2.24 ± 2.32	64.4	5.60 ± 3.49	55.4	<0.001
6month after MWA	1.24 ± 1.45	92.0	3.95 ± 2.67	68.7	<0.001
12month after MWA	0.22 ± 0.30	98.2	1.95 ± 1.67	85.9	<0.001
Last follow-up	0.03 ± 0.04	99.8	0.43 ± 0.52	97.3	<0.001

Values are presented as mean ± SD or percentage.

MWA, microwave ablation.

## Discussion

In this study, we retrospectively analyzed the outcomes of 41 predominantly cystic thyroid nodules that underwent MWA by two procedures.

Needle tract ablation was performed before aspirating cyst fluid in Group A. This method blocked the blood vessels on the capsule wall and prevented intracystic hemorrhaging caused by the penetration of the puncture needle. MWA was performed directly after aspirating liquid of the nodules in Group B. When the puncture needle penetrated into the nodule, it was easy to puncture the blood vessel of the capsule wall, causing intracystic hemorrhaging (13). Overall, our results demonstrate that the therapeutic effect of MWA in Group A is superior to that of Group B. Not surprisingly, our results showed that the incidence of intracystic hemorrhage in Group B was 40%. Compared with previous studies (4, 8, 15, 18), the probability of intracystic hemorrhage was extremely high. The main reasons for our finding were as follows: First, these nodules with intracystic hemorrhage were large in mean volume (V=19.5 ml) and high in capsule wall tension. Second, nodules with intracystic hemorrhage were richer in blood supply. Among the eight intraoperative intracystic hemorrhaging nodules, five (62.5%) nodules had an abundant blood supply. Among the nodules with abundant blood supply, the probability of intraoperative intracystic hemorrhaging in Group B was greater than that in Group A (57.1% *vs.* 8.3%), indicating that prior needle tract ablation can reduce the incidence of intraoperative intracystic hemorrhaging, but the difference was not statistically significant, which may be affected to some extent by the limited sample size.

Intracystic hemorrhage increased the complexity of the intervention, albeit the intervention time for each nodule was longer. Accordingly, the length of hospital stay was increased. In our study, three patients in Group A were hospitalized for 2 days (2 patients with intracystic hemorrhage during MWA and one patient with hematoma formation). Six patients in Group B were hospitalized for 2 days due to intraoperative intracystic hemorrhage, and two of them had hematoma after MWA. Patients in Group B had more hospitalization days than those in Group A, but the difference was not statistically significant (P=0.085). This may be related to the limited number of patients.

Minor complications were observed in two patients after MWA in Group B, with an incidence of 13.3% (2/15). Both of them had compression symptoms due to hematoma but fully recovered after 1 month. The minor complication rate was 4.3% (1/23) in Group A, consisting of mild pain after MWA that resolved within a few hours. The incidence of minor complications in Group B was higher, although not statistically significant. In addition, no major complications, such as voice changes, skin burning, infection, esophageal or tracheal injury, were observed among the patients. Therefore, the adverse effects are negligible. Some complications have been reported after thermal ablation, such as voice changes, skin burns, and delayed abscess formation (19, 20). Therefore, we stipulate that thyroid nodules be routinely admitted to the hospital for observation at least 24 h after MWA, and if there is intracystic hemorrhage during MWA or hematoma formation after MWA, additional observation is performed until the symptoms are relieved.

Consistent with the literature (12, 21–23), MWA was effective in treating benign TNs, as it induced a significant reduction in nodule volume, improved clinical symptoms, caused fewer complications, guaranteed quick recovery, met patients’ aesthetic needs, and showed less physiological and psychological interference. Our results showed that the VRR rapidly increased within the first 3 months and then increased steadily later on, which was consistent with other studies (13, 24).

MWA is a newly developed local thermal ablation technique that has a fast heating speed, strong coagulation ability, and a large ablation zone and has become a great therapeutic method in heat ablation therapy. Compared with RFA (radiofrequency ablation), LA (laser ablation), and HIFU (high-intensity focused ultrasound), MWA has the advantages of high energy generation, a large ablation area, a short treatment time, less influence from heat sink effects, and complete tumor inactivation (25, 26).

However, our study still had several limitations. First, the existence of thyroid function and autoimmune diseases was excluded in our work, and whether intracystic hemorrhage during MWA is related to these factors was uncertain. Second, there was an absence of histological confirmation of TNs, similar to other studies without surgery. Therefore, malignant nodules cannot be completely ruled out. In this regard, we intend to consider obtaining pathologic findings in the subsequent study. Last but not least, the retrospective study was designed with relatively small samples, and the performance was investigated at a single center. Future prospective multicenter studies are necessary to confirm our results.

## Conclusion

In summary, the results of our study indicate that ablating the aspiration tract prior to cyst fluid aspiration in MWA for predominantly cystic thyroid nodules avoids intracystic hemorrhaging, provides better safety and efficacy, and is worthy of wider use and clinical application.

## Data Availability Statement

The raw data supporting the conclusions of this article will be made available by the authors, without undue reservation.

## Ethics Statement

The studies involving human participants were reviewed and approved by the Ethics Committee of the Affiliated Hospital of Jiangsu University. The patients/participants provided their written informed consent to participate in this study.

## Author Contributions

HY contributed to collecting the data. HY and YC were responsible for writing and editing the manuscript. BC, KW and ZC performed the operation. SZ, ZZ, HF and MA reviewed the manuscript. All authors contributed to the article and approved the submitted version.

## Funding

This work was supported by the “169” Project of Zhenjiang City (YLJ201931), the Doctoral Start-up Fund of Affiliated Hospital of Jiangsu University (jdfyRC2019005) and the Social Development Program of Zhenjiang City (SH2019038).

## Conflict of Interest

The authors declare that the research was conducted in the absence of any commercial or financial relationships that could be construed as a potential conflict of interest.

## Publisher’s Note

All claims expressed in this article are solely those of the authors and do not necessarily represent those of their affiliated organizations, or those of the publisher, the editors and the reviewers. Any product that may be evaluated in this article, or claim that may be made by its manufacturer, is not guaranteed or endorsed by the publisher.
